# Response to “follicular cystitis, a rare diagnosis in an emergency setting: A case report and literature review

**DOI:** 10.1016/j.ijscr.2025.111131

**Published:** 2025-03-08

**Authors:** Vilas Sabale, Onkar Sangha

**Affiliations:** Department of Urology, Dr D.Y. Patil Medical College, hospital & Research Centre, Pimpri, Pune, India

Dear Editor,

I recently read with great interest the article titled “ Follicular Cystitis, a Rare Diagnosis in an emergency setting: A case report and literature review.” published in International Journal of Surgery Case Reports, and I would like to offer a point of technique that could further contribute to the effective management of bladder clots during irrigation. (See Fig. 1, Fig. 2.)

**Fig. 1:**
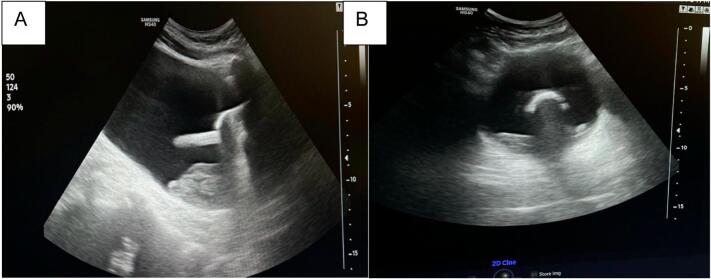
(A,B)- Ultrasonography of bladder in showing tip of foley catheter sitting over the debris with balloon inflated with air and water.

**Fig. 2:**
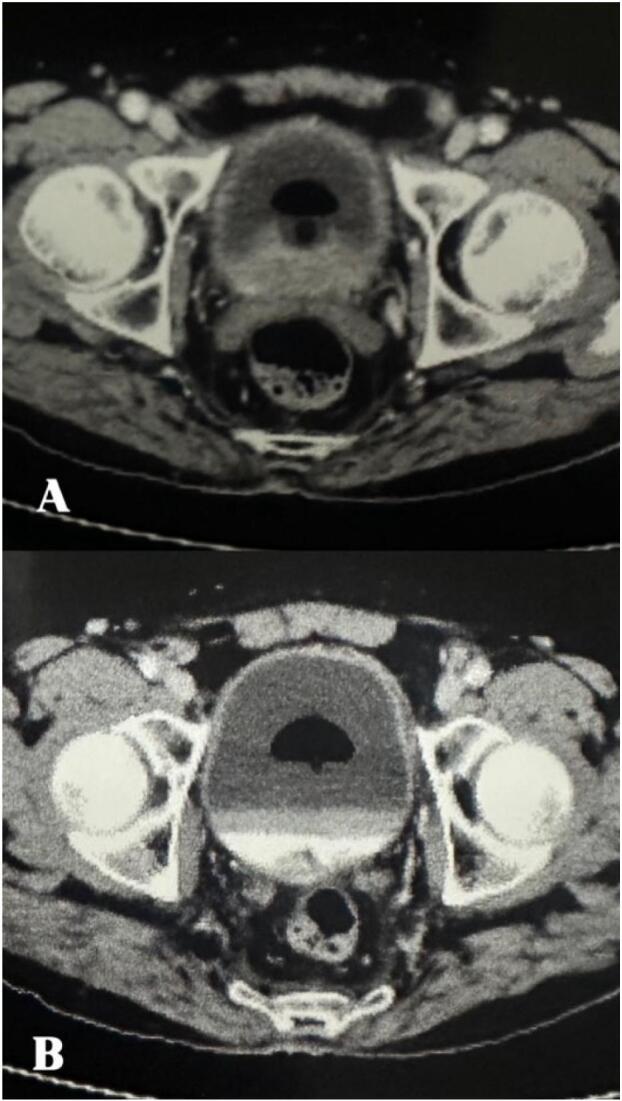
(A,B)- Contrast enhanced CT scan of bladder showing bubl of foley above the clot and debris.

As the article highlights, continuous bladder irrigation (CBI) with saline was used for preventing clot obstruction and managing haematuria. While these traditional methods remain the standard of care, I would like to propose a modification to the Foley catheter balloon inflation technique that has shown promise in specific clinical settings in our experience.

In cases where clots are small to medium in size and may not fully obstruct the urinary tract but still cause significant discomfort or partial obstruction, I have found that inflating the Foley balloon with **10** **cc of air** and **10** **cc of sterile water** can be an effective approach to promoting clot clearance.

This technique causes the foley balloon to float over the clots and urine with the tip of the draining catheter above the clots and urine at all times. This prevents recurrent blockage of catheter and leads to efficient continuous bladder irrigation.

Though this technique has not been widely studied in large-scale trials, it has shown favourable outcomes in the clinical settings where I have applied it.

I believe that including this modification in future studies of bladder clot and haematuria management could offer valuable insights into **enhancing the efficacy** and **safety** of non-invasive treatments. I look forward to seeing further advancements and discussions in this area, as this is a key aspect of bladder management that directly impacts patient comfort and outcomes.

Thank you for considering this contribution to an important topic. I look forward to your thoughts and any future discussions regarding innovations in bladder irrigation techniques.

Sincerely,

Onkar Singh Sangha

Senior Resident,

Department of Urology

Dr. D.Y. Patil Medical College, hospital and Research Centre, Pimpri, Pune, India

+ 919,814,239,994.

onkarsangha1304@gmail.com

## Table file

No table in submission for the letter to editor in response to Response to “Follicular Cystitis, a Rare Diagnosis in an emergency setting: A case report and literature review.”

## Consent

Written consent was taken from patient for publication. A copy is available for review on request.

## Ethical approval

Ethical approval not required.

## Funding

No funding for the letter to editor.

## Author contribution

Dr. Vilas Sabale^1^- Study concept, writing the paper, guidance on point of technique.

Dr. Onkar Sangha^2^- Study design, paper writing, corresponding author, performing procedure.

## Guarantor

Dr. Onkar Sangha.

## Research Registration Number

N/A.

## Conflict of interest statement

None.

